# Defoliation‐induced compensatory transpiration is compromised in *SUT4*‐RNAi *Populus*

**DOI:** 10.1002/pld3.268

**Published:** 2020-09-28

**Authors:** Scott A. Harding, Christopher J. Frost, Chung‐Jui Tsai

**Affiliations:** ^1^ Warnell School of Forestry and Natural Resources Department of Genetics and Department of Plant Biology University of Georgia Athens GA USA; ^2^ Present address: BIO5 Institute University of Arizona Tucson AZ 85719 USA

**Keywords:** defoliation, relative water content, subcellular sucrose partitioning, water uptake

## Abstract

The tonoplast sucrose transporter PtaSUT4 is well expressed in leaves of *Populus tremula* × *Populus alba* (INRA 717‐IB4), and its inhibition by RNA‐interference (RNAi) alters leaf sucrose homeostasis. Whether sucrose partitioning between the vacuole and the cytosol is modulated by PtaSUT4 for specific physiological outcomes in *Populus* remains unexplored. In this study, partial defoliation was used to elicit compensatory increases in photosynthesis and transpiration by the remaining leaves in greenhouse‐grown poplar. Water uptake, leaf gas exchange properties, growth and nonstructural carbohydrate abundance in source and sink organs were then compared between wild‐type and *SUT4*‐RNAi lines. Partial defoliation increased maximum photosynthesis rates similarly in all lines. There was no indication that source leaf sugar levels changed differently between wild‐type and RNAi plants following partial defoliation. Sink levels of hexose (glucose and fructose) and starch decreased similarly in all lines. Interestingly, plant water uptake after partial defoliation was not as well sustained in RNAi as in wild‐type plants. While the compensatory increase in photosynthesis was similar between genotypes, leaf transpiration increased less robustly in RNAi than wild‐type plants. SUT4‐RNAi and wild‐type source leaves differed constitutively in their bulk modulus of elasticity, a measure of leaf turgor, and storage water capacitance. The data demonstrate that reduced sucrose partitioning due to *PtaSUT4*‐RNAi altered turgor control and compensatory transpiration capacity more strikingly than photosynthesis and sugar export. The results are consistent with the interpretation that SUT4 may control vacuolar turgor independently of sink carbon provisioning.

## 1 INTRODUCTION

Sucrose and its hexose breakdown products contribute significantly to the high osmolalities often found in leaves of temperate latitude tree species including *Populus* (Rennie & Turgeon, 2009; Slewinski, Zhang, & Turgeon, 2013). High osmolality in the mesophyll supports leaf turgor in trees, and is also thought to reflect the operation of a subcellular partitioning mechanism that effectively concentrates sucrose into the cytosol for passive diffusion toward the phloem (Fu, Cheng, Guo, & Turgeon, 2011). Sucrose then enters the minor phloem through plasmodesmata for hydrostatically driven export to distant sinks (Carvalho, Turgeon, Owens, & Niklas, 2017; Davidson, Keller, & Turgeon, 2011; Fu et al., 2011; Liesche, 2017; Zhang et al., 2014). The differential partitioning of mesophyll sucrose has long been presumed to involve the central vacuole, but at the same time the amount of partitioning theoretically required to drive passive diffusion into the phloem could be minimal (Turgeon & Medville, 1998). RNAi inhibition of the class III tonoplast sucrose symporter in *Populus tremula* × *alba* (INRA 717‐IB4), *PtaSUT4*, increased source leaf sucrose levels, but has essentially no negative effect on shoot growth or sugar concentrations in sink organs under normal growth conditions (Payyavula, Tay, Tsai, & Harding, 2011). Based on the known biochemical function of class III tonoplast SUTs, elevated sucrose level is probably due to vacuolar sequestration (Julius, Leach, Tran, Mertz, & Braun, 2017; Payyavula et al., 2011; Reinders, Sivitz, Starker, Gantt, & Ward, 2008). Photosynthesis is not changed in the *SUT4*‐RNAi poplars (Frost, Nyamdari, Tsai, & Harding, 2012). This together with the absence of an effect on shoot biomass indicate that increased vacuolar sequestration in *SUT4‐*RNAi poplar has at most a minor effect on overall plant carbon budget. Although a 5%–10% reduction in root:shoot ratio is sometimes observed in the poplar RNAi lines, this occurs in a context of sugar level increases in stem sinks stress (Frost et al., 2012; Payyavula et al., 2011; Xue, Frost, Tsai, & Harding, 2016).

Class III tonoplast SUT4 orthologs have been studied in a number of species, and besides *Populus*, their role in sucrose export has been most thoroughly addressed in rice and maize. Rice (*Oryza sativa*) mutants defective in class III *OsSUT2* exhibit severely reduced plant growth and yield (Eom et al., 2011; Eom, Choi, Ward, & Jeon, 2012). Sucrose hyperaccumulation in mutant source leaves led the authors to conclude that sucrose can follow a symplastic route to the phloem which was blocked in the *ossut2* mutants (Eom et al., 2011, 2012). Similarly, in maize (*Zea mays*), mutation of class III *ZmSUT2* resulted in leaf sucrose hyperaccumulation and severely reduced growth, but tracer analysis showed that sucrose export from source leaves of the maize mutants was not impaired (Leach, Tran, Slewinski, Meeley, & Braun, 2017). Accordingly, day versus night sucrose levels oscillated with the same amplitude in the *zmsut2* mutant and the wild type (Leach et al., 2017). In fact, diurnal oscillations in leaf sucrose content have not yet been shown to involve the vacuolar pool. The combined evidence clearly suggests the importance of tonoplast SUT to leaf sucrose homeostasis, but less clearly to sucrose export from source leaves, either in active or passive phloem‐loading species. The situation in rice is presently unresolved even though phloem loading and SUT involvement do not appear to differ from maize (Eom, Nguyen, Lee, Lee, & Jeon, 2016; Julius et al., 2017). Regardless, it is interesting to note that class III *SUT* expression begins to increase at midday, and to peak in the early evening in maize and potato (Chincinska et al., 2008; Leach et al., 2017). While this may presage nighttime sucrose export to sinks, it also overlaps with the midday depression of photosynthesis due to diurnal vapor pressure deficit stress (Koyama & Takemoto, 2014; Singsaas, Ort, & Delucia, 2000). The discussion about tonoplast SUT function to date has focused more on sucrose export to sinks than on possible roles in osmotic control or stress amelioration.

Vacuolar uptake is mediated by tonoplast sugar proton antiporters (TSTs), and efflux into the cytosol by tonoplast SUT4, among others (Hedrich, Sauer, & Neuhaus, 2015; Reinders et al., 2008; Schneider et al., 2012; Schulz et al., 2011). In leaves of *Arabidopsis, AtSUT4* is very weakly expressed, representing less than 5% of leaf *SUT* transcript abundance (Lloyd & Zakhleniuk, 2004; Schneider et al., 2012). In most other studied species, the *SUT4* ortholog represents a much larger percentage of overall *SUT* gene expression (Xu, Chen, Ren, Chen, & Liesche, 2018). It may be relevant to the eventual interpretation of SUT4 function that published leaf sucrose levels are an order of magnitude lower in *Arabidopsis* than in poplar, maize and rice (Eom et al., 2011; Leach et al., 2017; Payyavula et al., 2011; Srivastava, Ganesan, Ismail, & Ayre, 2008). Thermodynamically, sucrose export from the vacuole would be more uphill in the other species than in *Arabidopsis* since sucrose concentrations are generally much higher in the cytosol than the vacuole (Nadwodnik & Lohaus, 2008). It is not necessarily surprising therefore that a significant role for foliar SUT4, an ATP‐dependent transporter, in growth control or stress amelioration in *Arabidopsis* has not been demonstrated (Durand et al., 2016; Gong et al., 2015).

Interestingly, in rice, *OsSUT2* promoter activity also is not induced by a range of abiotic stressors, including drought (Eom et al., 2011). In the poplar clone 717, however, *PtaSUT4* transcript levels vary with water availability, remaining low (50% lower than control) in wild type (WT) leaves that expand under chronic mild drought conditions, while increasing in leaves and other organs of *SUT4*‐RNAi and WT plants subjected to an acute drought (Frost et al., 2012; Xue et al., 2016). The increases were most striking in RNAi plants with low basal expression of *PtaSUT4* (Xue et al., 2016). A *SUT4* increase has been reported in poplar stems after a severe drought treatment (Frost et al., 2012; Pagliarani et al., 2019; Xue et al., 2016). In addition, aquaporin expression differs between drought‐stressed WT and *SUT4*‐RNAi poplars, consistent with a link between sucrose trafficking and changes in water flux (Xue et al., 2016). The dearth of evidence to support a role for SUT4 orthologs in sucrose export from source leaves (Durand et al., 2016; Julius et al., 2017; Leach et al., 2017; Payyavula et al., 2011), along with evidence for a possible hydraulic role in stress amelioration in *Populus* warrant the further investigation of PtaSUT4 function.

As large, long‐lived perennials, broadleaf tree species constantly undergo changes in source:sink ratio which trigger changes in photosynthesis, water uptake and carbohydrate utilization as plants adjust (Turnbull, Adams, & Warren, 2007; Wiley, Huepenbecker, Casper, & Helliker, 2013). Compensatory increases in photosynthesis and transpiration in the absence of other stresses have been observed following partial defoliation or stand thinning (Breda, Granier, & Aussenac, 1995; Eyles et al., 2013). Diurnal oscillations in leaf water potential, hydraulic conductivity, and transpiration, even under well‐watered conditions have also been reported (Simonin et al., 2015). The present work utilized pot‐grown WT and *SUT4*‐RNAi saplings to assess whether PtaSUT4 capacity is a factor in compensatory photosynthesis and transpiration following reduced source:sink ratio by partial defoliation. Partial defoliation comprises a milder and more easily controlled means of manipulating source:sink ratio and plant water use than drought, the response to which is known to have complex genetic underpinnings in *Populus* (Tschaplinski et al., 2006). The effects of *SUT4*‐RNAi on the leaf hydraulic properties, modulus of elasticity (ε) and storage water capacitance (C) were also examined since they may reflect on solute partitioning and a link between SUT4 function and observed responses.

## 2 MATERIALS AND METHODS

### 2.1 Plant propagation and growth

Generation and characterization of the *SUT4*‐RNAi lines in the hybrid poplar *P. tremula* × *P. alba* INRA 717‐1B4 background (717) were previously described (Payyavula et al., 2011; Xue et al., 2016). RNAi lines F and G were utilized for the current study (Payyavula et al., 2011). The plants grow normally under low‐stress greenhouse conditions and exhibit approximately 45% and 30% residual *PtaSUT4* expression in partially and fully expanded leaves, respectively, in line F; and 20% residual expression in all leaves of line G. Single‐node cuttings were grown in a glasshouse in 4 gallon tree pots (Hummert International) containing commercial soil mixture (Fafard 3B) supplemented with Osmocote (15‐9‐12 NPK 4‐month release) as previously described (Frost et al., 2012; Xue et al., 2016). Two experiments were conducted, the first, an 8‐day experiment during May and June 2012; the second, a 16‐day experiment during May and June 2016. The second experiment was necessitated because samples from the first experiment became degraded due to freezer malfunction before starch, condensed tannin and phenolic glycosides were measured. Outdoor conditions were essentially identical for both experiments. In both cases, evaporative cooling pads kept daytime temperatures below 35°C at canopy height. Plants were watered daily throughout the growth and treatment phases of the experiments.

### 2.2 First defoliation experiment

For the first experiment, sixteen vegetatively propagated copies of WT and two RNAi lines (F and G) of roughly uniform height (~1.5 m) were randomly assigned to three defoliation treatments and a control treatment with no defoliation (*N* = 4 plants per genotype x treatment combination). At the time of defoliation, upper stems were marked at the position of leaf plastochron index zero (LPI‐0) according to established developmental index criteria for *Populus* (Larson & Isebrands, 1971). For the 25% and 50% partial defoliation treatments, respectively, every fourth or every other leaf and its associated bud scale below LPI‐0 was removed. For the 100% defoliation treatment, all leaves below LPI‐0 were removed. Outside conditions were sunny and warm during the treatment period with plant heights increasing 4–5 cm/day in 0%, 25% and 50% defoliation treatments; and 2–3 cm per day for the 100% defoliation treatment. Experimental plants were maintained with normal watering for 8 days at which time photosynthetic parameters were measured and tissue harvesting was carried out as detailed below.

### 2.3 Photosynthesis

Leaf photosynthesis, stomatal conductance and transpiration of WT and *SUT4*‐RNAi transgenic plants were determined at midday using a Licor LI‐6400XS (LiCor, Lincoln, NE) as described previously (Frost et al., 2012). LPI‐6, a 75% expanded source leaf, was used for the measurements. Approximately, 12 new leaves, counting down from LPI‐0, were observed on the new growth after the start of the treatment period. The LPI‐6 assayed at day 8 was not yet emerged (approximately LPI minus‐5) when treatments began. Photosynthesis, stomatal conductance and transpiration rates were determined at a single, saturating light intensity of 1,500 µmol m^−2^ s^−1^.

### 2.4 Biomass analysis and tissue sampling

Each plant comprised a single unbranched stem. No syleptic bud release along the stem occurred during the treatment phase. Immediately, prior to harvest, a fully expanded source leaf at LPI‐10 was weighed and snap frozen in liquid nitrogen for metabolite analysis. A stem section between LPI‐15 and 20 was debarked after weighing, and both bark and wood fractions snap frozen for metabolite analysis. Upon harvest, fresh weights of the remaining leaves and stems were obtained. Tissues were then placed in a forced‐air oven and dried at 100°C for 48 hr for dry weight determinations.

### 2.5 Sucrose and hexose analysis

Snap‐frozen leaves were lyophilized for 48 hr (FreeZone 2.5, Labconco), then, ground through a 40 mesh sieve using a Wiley Mill (Thomas Scientific). Aliquots of the coarse powder were further ball‐milled in a Mini Bead‐beater (Biospec 3110Bx) at intensity setting 25 for two cycles. Ten mg of the lyophilized powder was suspended in a microtube with 500 µl methanol:chloroform (1:1, v/v) containing adonitol as internal standard, and sonicated for 15 min in a sonic bath with pre‐chilled water (4°C). Deionized water (200 µl) was then added to the tubes and samples vortexed and re‐sonicated for 5 min. After centrifugation, 10 µl of the upper aqueous‐methanol phase was evaporated to dryness in 200 µl glass microserts, and derivitized for Gas Chromatography‐Mass Spectrometry (GC‐MS) as described (Jeong, Jiang, Chen, Tsai, & Harding, 2004). Briefly, the dried extract was methoximated in 15 µl methoxyamine hydrochloride/pyridine solution (20 mg/ml; Sigma‐Aldrich) for 30 min at 30°C, then, silyated for 90 min at 60°C after adding 30 µl N‐Methyl‐N‐(trimethylsilyl) trifluoroacetamide (Sigma‐Aldrich). Incubations were carried out in a Vortemp 56 orbital shaker (Labnet) at 600 rpm. Derivitized samples were injected (1 µl) in 25:1 split mode at an inlet temperature of 250°C. Metabolites were resolved on a DB‐5MS column (30 m length, 0.25 mm diameter with DuraGuard pre‐column) with a helium flow of 1 ml/min. GC (Agilent 7890A) oven temperature at injection was 80°C. Following a 1 min hold at 80°C, temperature was ramped 20°C/min to 200°C, then 10°C/min to 320°C with a 6.5 min hold at 320°C. Metabolites were detected using an Agilent 5975C MS with source and quadrupole mass filter temperature setting of 230°C and 150°C, respectively. Mass spectra were collected in scanning ion mode (*m/z* 50 and 500) in ChemStation (Agilent) and deconvoluted using AnalyzerPro (SpectralWorks). Peak retention times and spectral matches corresponding to fructose, glucose and sucrose were determined using authentic standards. Peaks areas were integrated using AnalyzerPro.

### 2.6 Second defoliation experiment

The second experiment was conducted mainly to collect tissues for metabolite assays (starch, condensed tannins and salicinoid phenolic glycosides) that were not carried out for the first experiment due to sample deterioration during long‐term storage. In addition, conducting the second experiment presented an opportunity to extend the defoliation treatment for further assessment of defoliation effects on growth. Only a 50% defoliation treatment was utilized, and the duration of the second experiment was increased to 16 days. Starch, condensed tannin and phenolic glycoside levels were measured in order to compare RNAi effects on growth with those on nonstructural metabolic sinks. Plants were propagated and grown as before and were approximately the same size at the start of treatment as in the first experiment. Tissue collection and processing for metabolic analysis followed the same procedures as above.

### 2.7 Starch

Ten mg of ball‐milled leaf, bark or stem powder in 2 ml Eppendorf tubes were extracted five times using 1.5 ml ethanol:chloroform (1:1, v/v) with sonication. After two rinses with 80% ethanol, α‐amylase (1,000 U, Sigma A4551) dissolved in 0.5 ml buffer (0.1 M sodium acetate, pH 5.0, with 5 mM CaCl_2_) was added to the extracted residue. After a 30 min incubation at 85°C with 800 rpm shaking, samples were cooled and 10 μl of sodium acetate buffer (pH 5.0) containing 5 U amyloglucosidase (Sigma A1602) was added to the digest. Samples were digested at 50°C for 48 hr with shaking (800 rpm). Digests were then centrifuged at 24,000 *g* and an aliquot of the supernatant was removed for GC‐MS detection of glucose as described above.

### 2.8 Condensed tannins

Condensed tannins (CT) were analyzed as in our previous work (Harding et al., 2005). Briefly, approximately 10 mg freeze‐dried tissue powder was extracted in 600 μl of methanol for 15 min in an ultrasonic bath and centrifuged at 15,000 *g* for 10 min. Pigment‐containing supernatant was combined with water (400 µl) and chloroform (400 µl) in a new tube, then, vortexed and centrifuged to remove pigments. The depigmented pellet and supernatant were combined and dried down for CT analysis by the butanol‐HCl method (Porter, Hrstich, & Chan, 1985). Following incubation of the residues at 95°C for 20 min in 1 ml butanol‐5% hydrochloric acid containing ferric ammonium sulfate, absorbance (A_550_) was read and quantified against aspen leaf CT standards.

### 2.9 Phenolic glycosides

Approximately, 10 mg ball‐milled, freeze‐dried tissue leaf or bark tissue was used for each assay. Powders were sonicated twice for 15 min each at 4°C in 400 µl of a master mix containing chloroform:methanol (1:1, v/v) and 700 µM D_5_‐benzoic acid internal standard. Water (200 µl) was then added to the sonicate which was vortexed and centrifuged to obtain a pigment‐free upper phase for HPLC‐TOF analysis. Samples were chromatographed using a Zorbax Eclipse XDB‐C18 High Resolution 4.6 × 50 mm column with 1.8 micron particle size. Mobile phases were (A) 97% H_2_O/3% acetonitrile/0.1% formic acid and (B) 97% acetonitrile/3% H_2_O/0.1% formic acid. Column temperature was 30°C. Elution followed the gradient: 3%B (0–1 min); 3%B‐17%B (1–3 min); 17%B (3–5 min); 17%B–60%B (5–9 min); 60%B‐98%B (9–11 min). Detection was by MS‐TOF (Agilent 6,220) in negative mode using electro‐spray ionization, capillary voltage 3500V, and fragmenter voltage set at 125V. Retention times and MS spectra were compared to those of salicortin (formic acid adduct *m/z* 470.4) and tremulacin (formic acid adduct *m/z* 573.5) isolated from bark tissue.

### 2.10 Water uptake

Water uptake was determined gravimetrically by changes in pot weight over a 90‐min uptake period shortly before plants were to be harvested. Water uptake was calculated on a leaf biomass basis. From previous work, specific leaf area (cm/g dry weight) did not differ between lines (Frost et al., 2012).

### 2.11 Relative water content experiment

Relative water content (RWC) was determined as (fresh weight‐dry weight)/(hydrated weight‐dry weight). Hydrated weight was obtained after allowing leaves to equilibrate in a dark chamber with petioles immersed in water for 4 hr. Additional equilibration did not measurably increase hydrated weight. Primary internode sections were floated on water for the 4‐hr hydration, then, blotted dry, weighed and dried in a forced air oven at 60°C for 48 hr.

### 2.12 Leaf pressure‐volume curve measurements

Storage water capacitance (C) and bulk modulus of elasticity (ε) were determined from pressure‐volume curves of fully expanded leaves (LPI 12–15) of undefoliated plants. Leaves were collected at 7 a.m. by razor excision at the base of the petiole. The end of the petiole was then submerged in distilled water and the distal 5 mm of the petiole sliced off under water. Leaves were then slipped into a Ziploc bag and allowed to equilibrate in the dark at room temperature for one hour. Pressure‐volume curves were constructed for each excised leaf by plotting the relationship between water potential (Ψw) and gravimetric water loss using a Schollander pressure bomb device and a balance with 0.1 mg precision. Between each Ψw measurement, leaves were weighed, returned to a darkened box and allowed to equilibrate for 20 min. Leaf dry weights were determined after 48 hr of drying in a 60°C forced air oven. Hydraulic parameters were determined from the curves according to established principals (Tyree & Hammel, 1972). Water storage capacitance, C, represents the change in relative water content (RWC) per unit change in Ψw. Modulus of elasticity, ε, represents the decrease in turgor (ΔP) per unit decrease in V (volume or water loss) or ΔP/ΔV. Values were obtained by curve extrapolations according to Meinzer, Woodruff, Marias, McCulloh, and Sevanto (2014). Leaves yielding higher ε values resisted water loss, keeping volume loss, ΔV, small, as pressure increased and Ψw leaf decreased (became more negative).

### 2.13 Transmission electron microscopy

Leaf tissue blocks were fixed in 2.5% glutaraldehyde, 0.1 M potassium phosphate pH 7.2, washed and postfixed in 1% osmium tetroxide, 0.1 M buffer for 2 hr at 4°C, dehydrated and embedded in Spurr's resin. Sections were cut at 60 nm using a Diatome diamond knife on a Reichert‐Jung Ultracut S ultramicrotome. Sections were picked up on grids and poststained using 4% uranyl acetate and lead citrate. Grids were observed in a JEOL JEM 1,011 transmission electron microscope operated at 80 kV.

## 3 RESULTS

### 3.1 Defoliation affected leaf photosynthesis similarly in WT and RNAi plants

Defoliation treatments were carried out to reduce source:sink ratio and examine *SUT4*‐RNAi effects on subsequent carbon and water use as plants adjusted. The leaf used for gas exchange measurements was LPI‐6 which emerged during the treatment period and was 75% expanded by day 8 (see Materials and Methods). The maximum photosynthesis rate (A_max_) of LPI‐6 did not differ between undefoliated and 25% defoliation plants for any of the genotypes, but trended higher in 50% defoliated plants of all lines compared to undefoliated controls (Table 1). While pooling of the data from all plant lines yielded an overall 15% A_max_ increase (*p* = .001), the increases were statistically significant only in RNAi line G, nearly so in line F, and not in WT (Table 1). Internal leaf CO_2_ concentrations trended lower in all lines as photosynthesis increased following 50% defoliation (Table 1).

**TABLE 1:** Leaf gas exchange after 8 days at various defoliation levels

Defoliation level	WT	line G	line F	overall
Maximum photosynthesis (mmol CO_2_ m^−2^ sec^−1^)				
0%	14.91 ± 0.67	12.39 ± 1.50*	13.53 ± 1.68	13.49 ± 1.64
25%	14.44 ± 1.16	13.51 ± 0.58	14.21 ± 1.25	14.05 ± 1.03
50%	16.16 ± 1.51	15.12 ± 0.34	15.38 ± 0.23	15.55 ± 0.94
100%	11.23 ± 0.59	12.46 ± 1.25	12.53 ± 0.10	12.07 ± 1.13
*p* (0% vs. 50%)	.248	**.012**	.071	**.001**
% increase	8.3%	22.0%	13.7%	15.3%
Transpiration rate (mmol H_2_O m^−2^ sec^−1^)				
0%	8.78 ± 0.60	9.18 ± 0.72	8.60 ± 0.87	8.86 ± 0.72
25%	9.65 ± 0.57	9.55 ± 1.20	9.44 ± 0.71	9.55 ± 0.79
50%	10.96 ± 0.77	10.33 ± 0.21	9.47 ± 0.37*	10.25 ± 0.82
100%	9.51 ± 0.58	9.40 ± 0.54	9.82 ± 0.27	9.61 ± 0.57
*p* (0% vs. 50%)	**.010**	**.047**	.115	**<.001**
% increase	24.8%	12.5%	10.1%	15.7%
Stomatal conductance rate (mmol m^−2^ sec^−1^)				
0%	0.80 ± 0.06	0.84 ± 0.10	0.71 ± 0.03	0.76 ± 0.13
25%	0.87 ± 0.04	0.91 ± 0.08	0.81 ± 0.10	0.86 ± 0.08
50%	0.78 ± 0.08	0.90 ± 0.08	0.76 ± 0.06	0.80 ± 0.09
100%	0.86 ± 0.13	0.89 ± 0.06	0.90 ± 0.10	0.88 ± 0.09
*p* (0% vs. 50%)	.665	.392	.246	.665
% increase	−3.0%	7.8%	7.2%	6.5%
Internal CO_2_ (ppm)				
0%	321.80 ± 2.69	330.25 ± 5.40*	323.00 ± 9.99	325.54 ± 7.09
25%	323.65 ± 6.51	328.80 ± 6.13	322.40 ± 7.04	324.95 ± 6.61
50%	311.10 ± 8.79	321.20 ± 0.72	315.60 ± 4.43	315.49 ± 6.83
100%	334.60 ± 5.40	332.60 ± 2.79	331.05 ± 3.52	332.75 ± 3.97
*p* (0% vs. 50%)	.102	**.037**	.236	**.004**
% increase	3.3%	2.7%	2.3%	3.1%

Note

Values represent the mean ± *SD* of *n* = 4 plants for each genotype, and *n* = 12 for overall. Statistical significance was determined by Student's *t* test between WT and RNAi plants (*p < .05) or between 0% and 50% treatments as indicated by *p* values (in bold).

### 3.2 Defoliation affected water uptake, transpiration and leaf water retention differently in WT and RNAi plants

**FIGURE 1:**
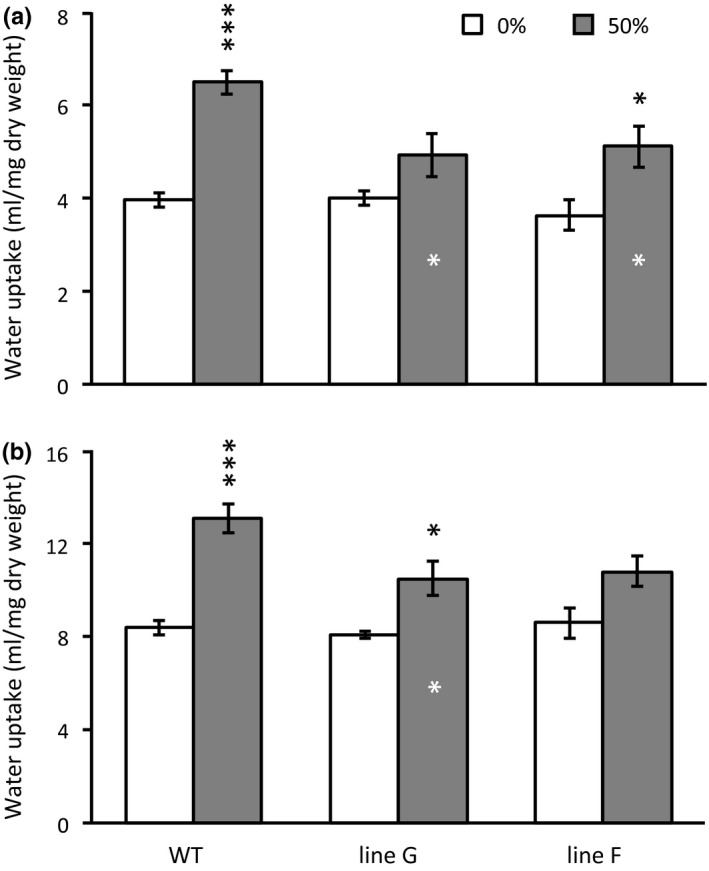
Partial defoliation increased plant water uptake on a leaf mass basis. Water uptake of greenhouse‐grown plants was measured gravimetrically over a 90‐min period at mid‐day (a), and for a separate cohort of plants over a 150‐min period at mid‐day (b). Conditions were sunny. Pots were watered to saturation 1‐hr before the measurement windows. Data represent the mean and standard error of *n* = 5 WT or *n* = 3 RNAi plants. Asterisks over bars indicate significant defoliation effects as determined by Student's *t* test, and asterisks inside the bar indicate significant differences between WT and RNAi lines at 50% defoliation (***p < .001, *p < .05)

LPI‐6 transpiration rates were higher overall in 50% defoliated than in non‐defoliated controls in Experiment 1 (Table 1). A suppressive effect was observed in *SUT4*‐RNAi lines, as the increase was larger in WT by roughly twofold (25% vs. 10%–13%, Table 1). The increase was significant for WT and for one of the RNAi lines. Stomatal conductance did not differ between genotypes or defoliation treatments (Table 1). Individual leaf transpiration was not measured in Experiment 2, but plant water uptake normalized to leaf mass was measured in that experiment and repeated in a separate cohort of plants that were defoliated and harvested two weeks after the first cohort. Specific leaf area, or area per unit dry mass, does not differ significantly between the lines used in these experiments (Frost et al., 2012). In both cohorts, water uptake was measured just before harvest at 16 days (Figure 1). Water uptake was similar in non‐defoliated WT and RNAi plants in both trials, but the increase following 50% defoliation was approximately twofold greater in WT than in plants of either RNAi line (Figure 1).

**FIGURE 2:**
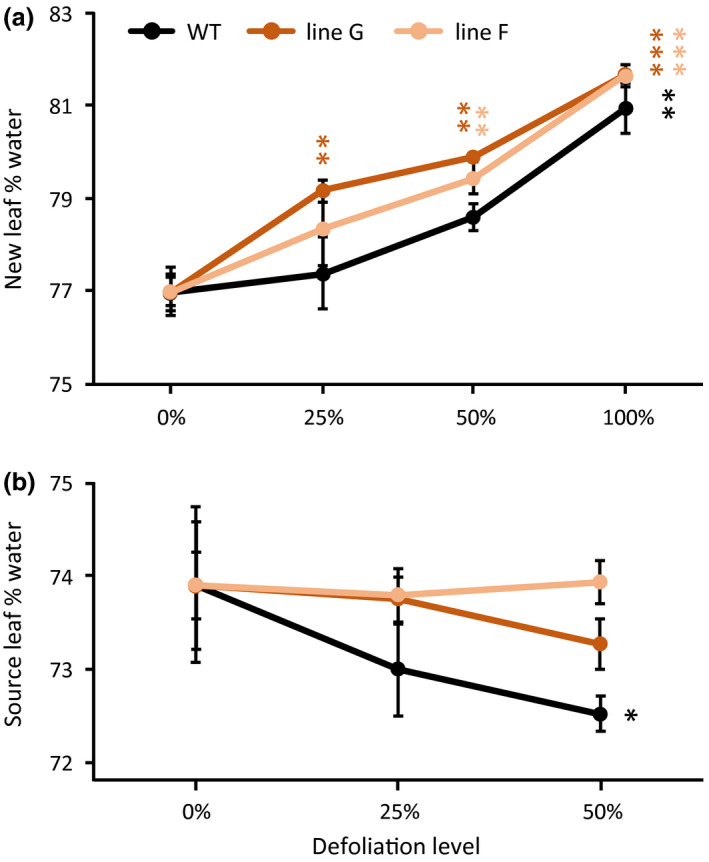
Leaf water contents responded differentially to partial defoliation in WT and RNAi plants. (a) New leaves that expanded during the defoliation treatment. (b) Source leaves that were fully expanded at the start of the defoliation treatment. The data points are connected by lines to depict the trend in water content change as defoliation levels increased for each plant line. Data represent the mean and standard error of *n* = 4 plants. Asterisks are color coded by plant line and indicate significant defoliation effects versus non‐defoliated (0%) controls as determined by Student's *t* test (***p < .001, **p < .01, *p < .05)

Leaf water contents were measured only in Experiment 1. Defoliation led to increased water content in new leaves that emerged and expanded after defoliation (Figure 2a). The increases were significant at all defoliation levels for the RNAi lines, but only at 100% defoliation for WT. At 100% defoliation, the percentage water increases were the same in all lines. Source leaves that were fully expanded before partial defoliations exhibited water content decreases in WT but not RNAi lines (Figure 2b).

### 3.3 Defoliation affected sucrose and hexose levels similarly in WT and RNAi plants

**FIGURE 3:**
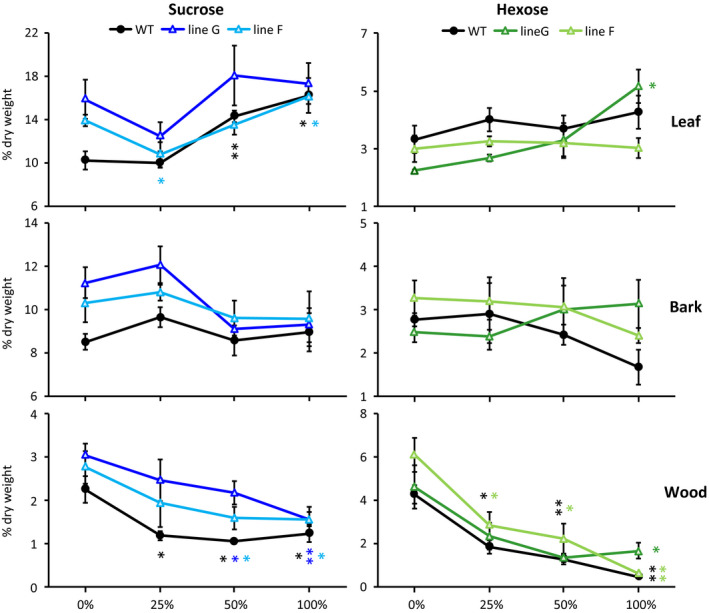
Sucrose and hexose concentration trends in leaves, bark and wood of WT and RNAi plants in Experiment 1. Tissues were harvested at mid‐day (12:00–14:00). Data represent the mean and standard error of *n* = 4 plants. Asterisks are color‐coded by line to indicate significant defoliation effects versus non‐defoliated (0%) controls as determined by Student's *t* test (**p <.01, *p < .05)

Prior to defoliation, sucrose levels were higher in the RNAi lines as has been reported (Payyavula et al., 2011). The 8‐day experiment tested three defoliation levels, 25%, 50% and 100%, and the only sustained trend across all three defoliation levels was decreasing soluble sugar contents (hexose and sucrose) in wood (Figure 3). The decrease was similar in WT and RNAi plants. Similar patterns were observed after a 16‐day 50% defoliation treatment (Experiment 2), with trends toward decreased soluble sugar levels in both wood and bark (Figure S1). The magnitude of the decrease after 50% defoliation (Experiment 1 and Experiment 2) or 100% defoliation (Experiment 1 only) differed little between lines (Figure 3 and Figure S1). The average sugar decreases after 8 and 16 days of partial (50%) defoliation are summarized in condensed form for all shoot organs to illustrate the broad finding that there was no clear net depletion of source leaf sugars by either treatment, but that depletions in the sink organs became more severe after 16 days for bark, and especially wood (Table 2).

**TABLE 2:** Overall changes in sucrose and hexose concentration in three shoot organs due to 8‐day or 16‐day defoliation treatments

	0%	50%	Fold change	*p*
8‐Day experiment				
Sucrose				
Leaf	13.4 ± 3.3	15.3 ± 3.8	1.15	.190
Bark	10.2 ± 1.7	9.1 ± 1.3	0.90	.120
Wood	2.7 ± 0.7	1.6 ± 0.6	0.59	**<.001**
Hexose				
Leaf	2.9 ± 0.8	3.4 ± 0.9	1.17	.221
Bark	2.9 ± 0.6	2.8 ± 1.0	0.99	.924
Wood	5.0 ± 1.7	2.8 ± 1.0	0.57	**.002**
16‐Day experiment				
Sucrose				
Leaf	13.7 ± 4.8	13.3 ± 4.4	0.97	.857
Bark	8.4 ± 1.6	6.3 ± 1.1	0.75	**.007**
Wood	3.7 ± 2.2	1.2 ± 0.5	0.33	**.004**
Hexose				
Leaf	1.6 ± 0.4	1.5 ± 0.3	0.95	.686
Bark	3.0 ± 0.7	1.8 ± 0.5	0.60	**<.001**
Wood	7.8 ± 1.4	2.8 ± 0.7	0.37	**<.001**

Note

Values represent the mean and standard deviation of *n* = 12 plants for each defoliation level. *p* values for defoliation effects were determined using Student's *t* test. See Figure 3 and Figure S1 for Individual line data. Bold values indicates the significance values (*p*).

**FIGURE 4:**
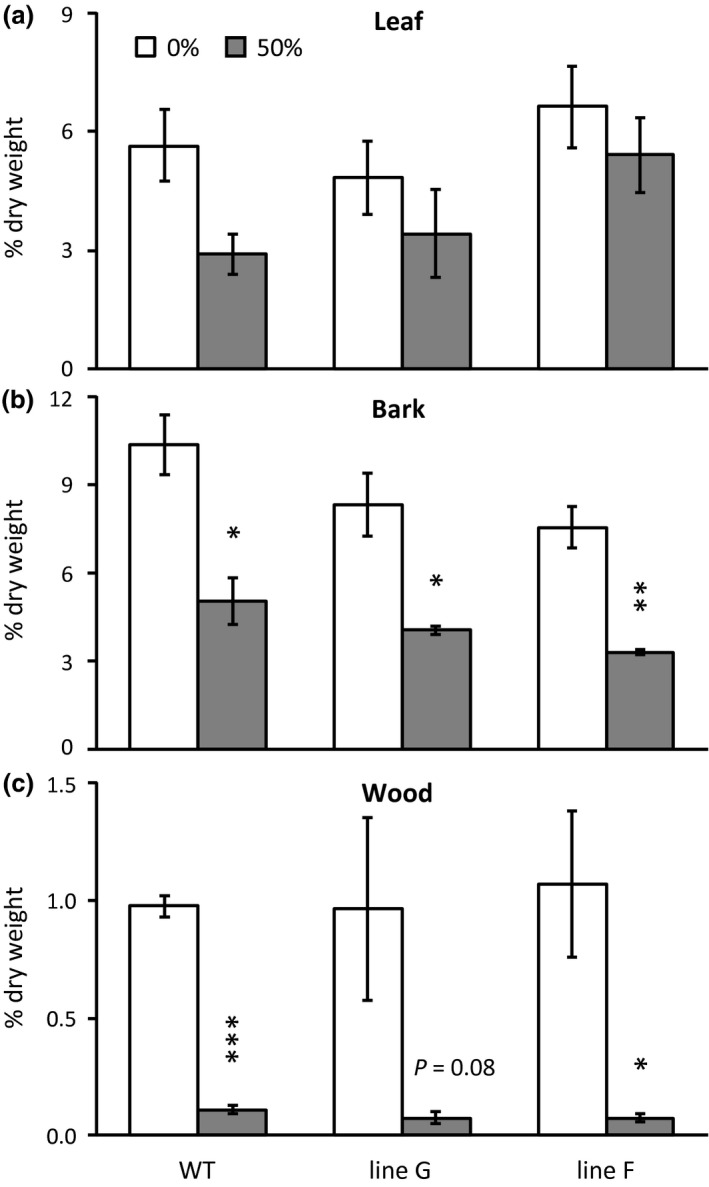
Defoliation‐induced starch loss in shoot organs in Experiment 2. Leaf (a); Bark (b); Wood (c). Tissues were harvested at mid‐day (12:00–14:00) under clear skies as in Experiment 1 (Figure 3). Data represent the mean and standard error of *n* = 3 plants. Asterisks indicate significant defoliation effects as determined by Student's *t* test (***p < .001, **p < .01, *p < .05)

Leaf, bark and wood starch were only measured in the 16‐day study (Experiment 2). By day 16, starch decreased, most clearly in bark and wood of the 50% defoliated plants (Figure 4). As with sugars, the decreases were similar in WT and RNAi lines (Figure 4).

### 3.4 Relative water content changed more rapidly in WT than RNAi tissues during solar warming

The transpiration and leaf water content differences between WT and RNAi plants led us to conduct a stand‐alone experiment to compare leaf relative water content (RWC) changes in WT and RNAi tissues during plant adjustment to increasing sunlight between 7 a.m. and 11 a.m. (Table 3). Water uptake was not measured but pots were watered to saturation at the start of the 4‐hr measurement period. As expected, water content decreased significantly in all lines as temperature and light intensity increased (Table 3). RWC also decreased significantly in all lines, but the decrease in upper stem internodes of RNAi was half that observed in WT plants between 7 a.m. and 11 a.m. (Table 3). While upper stem RWC did not differ between genotypes at 7 a.m., upper stem RWC was significantly higher in RNAi than WT plants at 11 a.m. During the same 4‐hr period, the RWC average in mature source leaves also decreased more in WT than RNAi plants (Table 3).

**TABLE 3:** Water content and relative water content changes in leaves and stem internodes during solar warming

	Water content (%)				Relative water content (%)			
	7 a.m.	11 a.m.	loss	*p*	7 a.m.	11 a.m.	loss	*p*
Internodes 5–6								
WT	79.00	76.30	2.70	**.025**	95.58	89.34	6.24	**.003**
RNAi	80.80	78.70	2.10	**.008**	96.47	93.29*	3.18	**.007**
LPI‐15								
WT	72.20	70.30	1.90	**.037**	98.68	93.40	5.28	**.001**
RNAi	69.80	68.50	1.30	**.022**	99.29	94.68	4.61	**<.001**

Note

Water content was determined as (fresh weight ‐ dry weight)/fresh weight, and relative water content was (fresh weight ‐dry weight)/(hydrated weight‐dry weight). Values represent the mean of *n* = 5 plants. Statistical significance was determined using Student's *t* test between WT and RNAi plants (*p < .05) or between measurement times (indicated by *p* values in bold).

### 3.5 Leaf hydraulic properties were altered in SUT4‐RNAi plants

**FIGURE 5:**
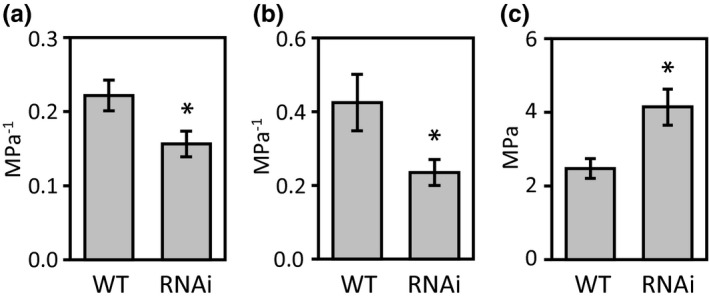
Leaf hydraulic parameters. Storage water capacitance before (a) and after (b) turgor loss, and bulk modulus of elasticity (c) were extrapolated from pressure‐volume curves. A fully expanded leaf, LPI‐12, was excised at 08:00 from undefoliated plants that were 1.5 m in height, then, equilibrated in sealed plastic bags in the dark for 1 hr prior to the analysis (see Materials and Methods). Bar heights represent the mean and *SEM* of *n* = 5 plants. Asterisks indicate significant difference as determined by Student's *t* test (*p < .05)

**FIGURE 6:**
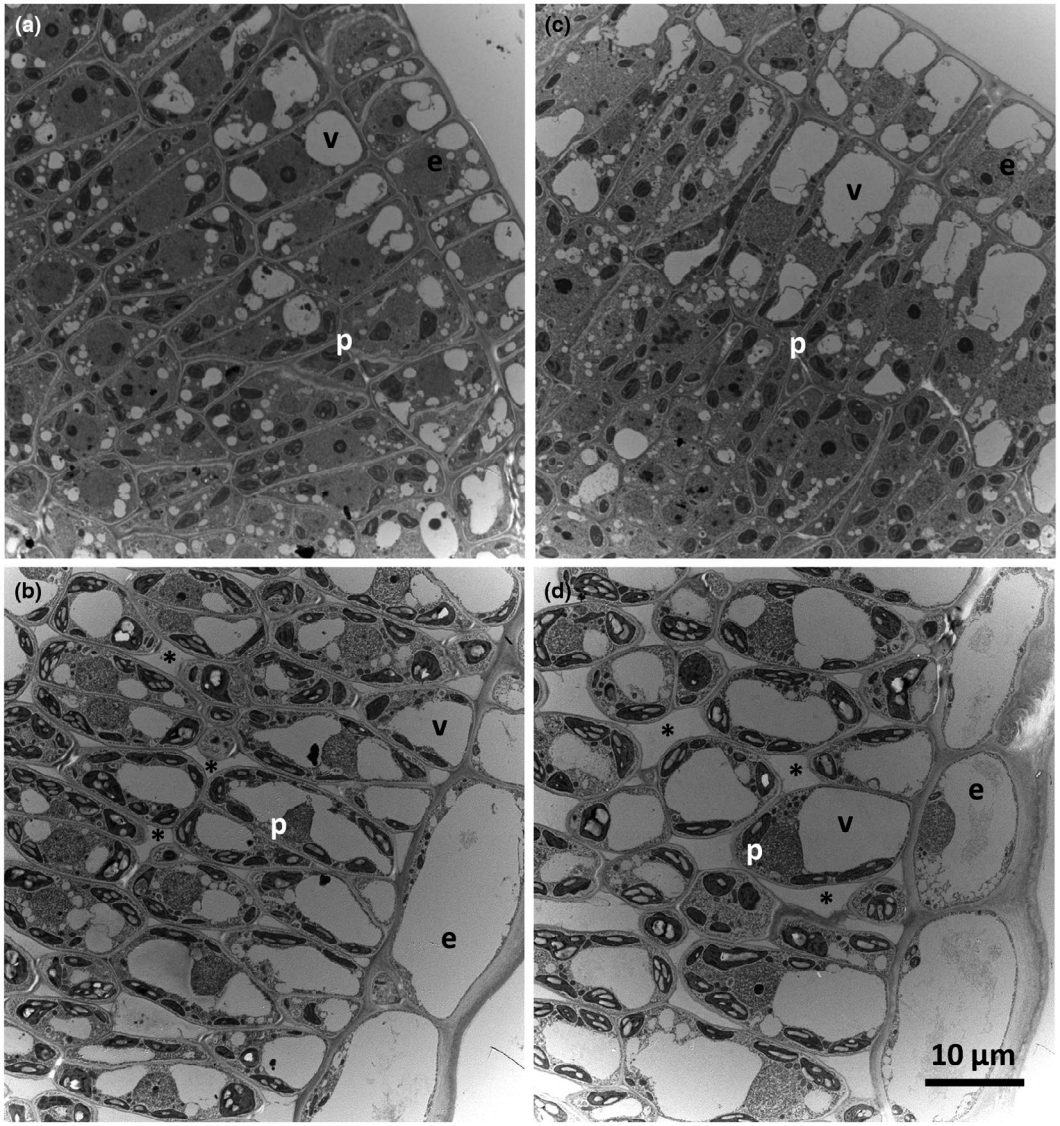
Subcellular morphology of expanding and expanded leaves. TEM images (3200X) of a one‐third expanded (a, c) and a fully expanded leaf (b, d) from WT (a‐b) and RNAi (c‐d) plants. e, epidermal cells; p, palisade mesophyll cells; v, vacuoles; asterisks denote intercellular gaps

In light of the RWC, transpiration, water uptake and leaf water content findings, a comparative examination of intrinsic leaf hydraulic properties using the approach of pressure‐volume curves was undertaken. Previous reports support a function for SUT4 orthologs in sucrose efflux from the vacuole (Endler et al., 2006; Leach et al., 2017; Payyavula et al., 2011), but there are no reports yet on whether partial loss of SUT4 function would have a measurable effect on intracellular turgor relationships. Pressure‐volume curves have long been used to relate turgor to osmotic potential via the analysis of changes in symplast volume as a function of increasing external pressure using a Schollander pressure bomb (Tyree & Hammel, 1972). The relationship between pressure and volume loss as indicated by storage water capacitance (C) and modulus of elasticity (ε) differed between WT and RNAi leaves from undefoliated plants (Figure 5). The assays were not undertaken for leaves of defoliated plants. Leaf mesophyll cell morphology of undefoliated plants was examined using TEM (Figure 6). Central vacuole size differed between WT and RNAi leaves, appearing larger in RNAi lines at both stages of leaf expansion. Cell packing appeared to be less well organized in the palisade mesophyll of fully expanded leaves (Figure 6).

### 3.6 Secondary metabolism was not altered in RNAi compared to WT plants

Nonstructural phenylpropanoid end‐products, including flavonoid‐derived proanthocyanidins, or condensed tannins (CT), and phenolic glycoside salicinoids typically accumulate in leaves and bark of poplars and other species of the family Salicaceae (Harding et al., 2005, 2014). They comprise important metabolic sinks because of their abundance, metabolic stability and impact on trophic interactions (Kandil, Grace, Seigler, & Cheeseman, 2004; Kleiner, Raffa, & Dickson, 1999; Ruuhola & Julkunen‐Tiitto, 2000). Average CT abundance trended lower, but not significantly, in leaves and bark of partially defoliated than of non‐defoliated plants, and there was no difference between genotypes (Table 4). Salicinoids, shown as the abundance of the major phenolic glycosides salicortin and tremulacin summed, exhibited no significant differences in leaves or bark in response to partial defoliation, though there were modest small decreases in bark. In all cases, the magnitudes of the CT and salicinoid trends were similar in WT and RNAi lines.

**TABLE 4:** Levels of salicinoids and condensed tannins in response to defoliation treatments

	Salicinoids			Condensed tannins		
	WT	Line G	Line F	WT	Line G	Line F
Leaf						
0%	8.00 ± 0.88	7.40 ± 0.39	7.83 ± 0.70	6.22 ± 3.28	6.54 ± 1.25	6.77 ± 0.80
50%	8.74 ± 1.22	7.74 ± 1.02	7.68 ± 0.95	3.44 ± 1.40	4.64 ± 1.81	5.49 ± 2.53
*p*	0.44	0.61	0.84	0.25	0.21	0.45
Bark						
0%	10.53 ± 0.75	10.97 ± 0.46	10.46 ± 0.57	2.80 ± 0.23	2.04 ± 0.25*	3.22 ± 0.75
50%	9.07 ± 0.60	9.25 ± 1.42	9.18 ± 1.33	2.33 ± 0.58	2.01 ± 0.50	2.18 ± 0.39
*p*	.06	.12	.3	.26	.95	.1

Note

Values represent the mean ± *SD* of *n* = 3 plants. Statistical significance was determined by Student's *t* test between WT and RNAi plants (*p < .05) or between 0% and 50% treatments shown by *p* values. Salicinoids represent the sum of salicortin and tremulacin levels.

### 3.7 Shoot growth was more negatively affected by partial defoliation in RNAi than WT

**FIGURE 7:**
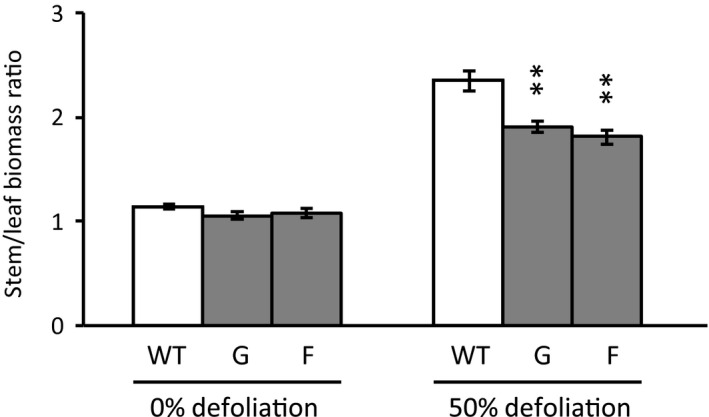
Stem growth per unit leaf mass following partial defoliation in Experiment 2. Tissues were harvested as in Figure 4. Plants were subjected to no defoliation or 50% defoliation for 16 days immediately prior to harvest. Data represent the mean and standard error of *n* = 5 WT or *n* = 3 RNAi plants. Asterisks indicate significant growth differences between partially defoliated WT and RNAi plants as determined by Student's *t* test (**p < .01)

Stem biomass was divided by leaf biomass to obtain an index of leaf ability to sustain stem biomass production, or “growth” in Experiment 2. Stem mass per unit leaf mass did not differ between WT and RNAi lines when intact plants were compared, but was greater for WT than either RNAi line when defoliated plants were compared (Figure 7).

## 4 DISCUSSION

### 4.1 PtaSUT4 and carbon

The knowledge gap we sought to address centers around the physiological role of PtaSUT4, a highly conserved tonoplast sucrose transporter with orthologs in monocots, dicots and basal angiosperm taxa (Peng, Gu, Xue, Leebens‐Mack, & Tsai, 2014). While SUT4 mediates sucrose efflux from the vacuole into the cytosol, the physiological relevance of that efflux in tree species such as *Populus* is being revealed only gradually (Frost et al., 2012; Payyavula et al., 2011; Xue et al., 2016). Based on reports from several species, there is little to support the notion that SUT4 orthologs somehow mediate sucrose export from source leaves to sink organs in tree or herbaceous species (Durand et al., 2016; Eom et al., 2016; Julius et al., 2017; Leach et al., 2017; Payyavula et al., 2011). While growth is severely reduced in null mutants of class III tonoplast SUT in rice and maize, sucrose export and long distance translocation are normal (Leach et al., 2017). ZmSUT2 has been suggested to mediate the nighttime export of sucrose from source leaves of maize, but the magnitude of the day‐night oscillation in leaf sucrose levels did not differ between WT and *zmsut2* nulls (Leach et al., 2017).

Results of the present study reinforce and extend our earlier findings (Frost et al., 2012; Payyavula et al., 2011) by showing that carbon fixation and sucrose export from leaves to shoot sinks responded similarly in WT and *SUT4*‐RNAi plants as source:sink ratio was decreased (Tables 1, 2, Figures 3, 4, and Figure S1). Sink levels of soluble sugars declined similarly in WT and RNAi plants in both defoliation experiments. Starch and soluble sugar contents were both measured in experiment 2, again with similar line‐to‐line trends in sink decreases. While sucrose levels were better sustained than starch in experiment 2 source leaves, no line‐to‐line differences were observed. Sampling was only performed at one time point, mid‐day, which means that the possibility of line‐to‐line differences in diurnal timing were not captured in these experiments. Source leaf sucrose levels were measured at dawn and just before dusk in one experiment with these lines and found to be stable (Payyavula et al., 2011). It is therefore possible that starch levels vary diurnally more than sucrose levels in *Populus* (Zhang et al., 2014). Levels of abundant defensive metabolites which do not exhibit diurnal oscillation due to their stability (Kandil et al., 2004; Kleiner et al., 1999; Ruuhola & Julkunen‐Tiitto, 2000) exhibited very similar levels and defoliation responses in WT and RNAi lines (Table 4).

### 4.2 PtaSUT4 and water

The defoliation treatments in this study were expected to increase transpiration, and thus, water flux through the remaining leaves as has been reported elsewhere for *Populus* (Liu, Eq uiza, Navarro‐Rodenas, Lee, & Zwiazek, 2014). Our earlier work with these lines provided some indication that water uptake is less well sustained by RNAi than WT plants as soil water is depleted (Frost et al., 2012). The defoliation treatment would therefore test leaf SUT4 function in a novel context with new physiological implications. In short, leaf water flux increased, as measured by transpiration or gravimetric water loss, but less robustly in RNAi than WT plants (Table 1 and Figure 1). Root conductivity was not measured in our study, but the greater increases we observed in RNAi leaf water content following defoliation (Figure 2) are not consistent with the idea of more limiting root hydraulic conductivity in the RNAi lines as can occur with partial defoliation (Liu et al., 2014). In light of the differences in water uptake, we conclude that the larger growth reductions in RNAi than WT plants after defoliation (Figure 7) were due primarily to lower compensatory water uptake.

Partial defoliation led to greater water retention by *SUT4*‐RNAi leaves than WT leaves, regardless of whether they expanded before or during defoliation (Figure 2a vs. b). A possible basis for this may relate to intrinsic differences between WT and RNAi lines in leaf hydraulic characteristics and mesophyll cell morphology (Figures 5, 6). TEM data from expanding and expanded leaves of undefoliated plants provide an indication that vacuole expansion was greater in the mesophyll of RNAi than WT leaves (Figure 6). In addition, turgor was better sustained under increasing external pressure in RNAi than WT leaves during the pressure‐volume assays, and this yielded higher ε values (Figure 5). The higher storage water capacitance (C) and lower modulus of elasticity (ε) values of WT leaves support the idea that *PtaSUT4*‐RNAi led to hydraulic changes in the mesophyll. Together, the TEM and pressure‐volume data are consistent with the idea that increased vacuolar turgor, presumably due to increased sucrose and water sequestration there, intrinsically promote greater vacuole expansion in RNAi lines. The tendency for expanding or expanded leaves of RNAi lines to have higher water contents than the corresponding WT leaves after partial defoliation may therefore derive from this intrinsic difference. While it is known that structural elements such as thickened cell walls or otherwise reinforced tissues within the leaf can impact C and ε, (Zwieniecki, Brodribb, & Holbrook, 2007), the most obvious differences between WT and RNAi leaves in the present study pertain to the vacuole. Specific leaf area, for example, has not been observed to differ between these lines (Frost et al., 2012), but might be expected to if there were structural differences such as thickened cell walls.

### 4.3 An integrative scenario

A scenario is offered to integrate the findings of this work. Partial defoliation would reduce source:sink ratio, and probably sucrose concentration in the cytosol due to a decrease in source capacity relative to the sink demand. Under those conditions, cellular ability to limit vacuole expansion in the remaining leaves would depend partly on export of vacuolar sucrose via SUT4. The sucrose release would have little enduring effect on leaf carbon export once the decrease in vacuole turgor was achieved. Impaired sucrose release in the case of *SUT4*‐RNAi could result in increased water uptake by the vacuole at the expense of cytosol volume. Loss of cytosolic volume and increased vacuolar sequestration of water due to high sucrose levels there would presumably interfere with extraxylary water movement through the mesophyll and toward sites of evaporation while favoring leaf water retention, as was observed. Any reduction in xylem water tension due to canopy removal would presumably contribute further to the observed leaf water content and transpiration differentials. Aquaporins facilitate the cross‐membrane dispersal of water via extraxylary apoplastic and symplastic spaces throughout the leaf, leading to enhanced transpiration. The idea that specifically localized tonoplast aquaporins can contribute to leaf hydraulic conductivity in this fashion has been proposed (Laur & Hacke, 2014). We have reported previously that RNAi‐silencing of *PtaSUT4* indeed influences the expression of several aquaporins during a drought response (Xue et al., 2016). The present work provides data to support the idea that SUT4 may function primarily to regulate vacuole turgor with consequences that may be more important for transpiration than for photosynthesis or sucrose export to sinks in poplar.

## CONFLICT OF INTEREST

The authors declare no conflict of interest.

## AUTHOR CONTRIBUTIONS

SAH and CJT conceived the study, CJF and SAH designed and performed the research, SAH analyzed the data and wrote the paper with contributions from CJF and CJT.

## Supporting information

Fig S1

Supplementary Material
