# Supplementary material for: Defoliation‐induced compensatory transpiration is compromised in SUT4‐RNAi Populus
Source: Plant Direct. 2020 Sep 28;4(9):e00268. doi: 10.1002/pld3.268 (PMC7522500; doi:10.1002/pld3.268)
Supplement: Supplementary file 1 — Fig S1 [file PLD3-4-e00268-s001.pdf]

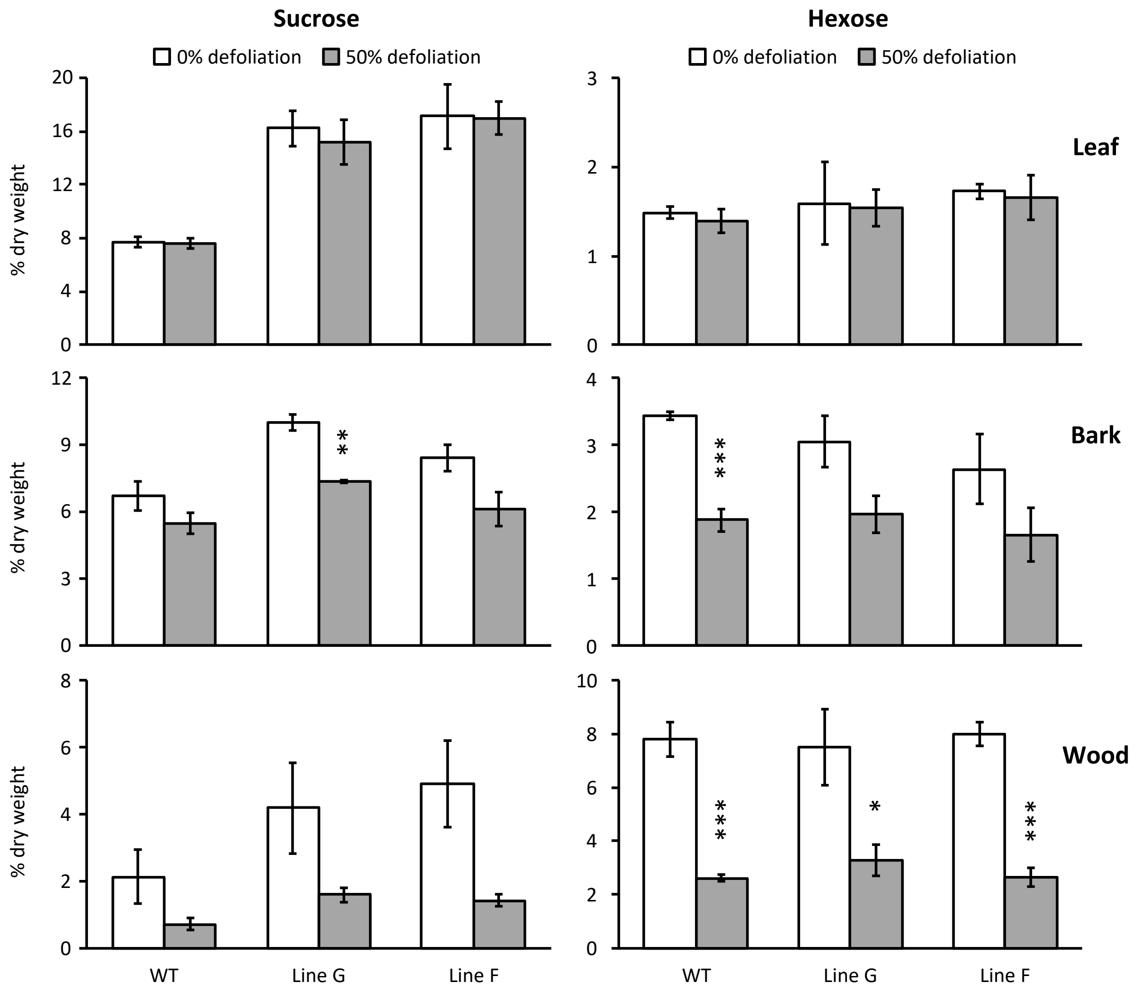

Supplemental Figure S1. Sucrose and hexose concentration trends in leaves, bark and wood of WT and RNAi plants in Experiment 2.

Data represent the mean and standard error of n=3 plants. Asterisks indicate significant defoliation effects as determined by Student's *t* test (*** $P < 0.001$, ** $P < 0.01$, * $P < 0.05$).
