# Supplementary material for: Defoliation‐induced compensatory transpiration is compromised in SUT4‐RNAi Populus
Source: Plant Direct. 2020 Sep 28;4(9):e00268. doi: 10.1002/pld3.268 (PMC7522500; doi:10.1002/pld3.268)
Supplement: Supplementary file 2 — Supplementary Material [file PLD3-4-e00268-s002.pdf]

Response to Review

Reviewer 1: I am confused by the LPI designations. Is the test leaf (LPI-6) the sixth leaf above the defoliated portion of the stem? In other words, is LPI-6 immature at the time of defoliation? It seems so since if it were below the apex, and mature at the time of defoliation, it would not be present in the 100% defoliation experiments. In line 106, LPI-6 is described as "newly expanded" but "newly" could refer to before or after defoliation. In any case it is unclear if the dash in LPI-6 is meant to be a minus sign.

Response: LPI-6 was immature at the time of defoliation. The LPI numbers represent the LPI position at the time of harvest 8 days after defoliation. This is now clarified in the Materials and Methods section (lines 132-135) as well as the Results section (lines 265-267).

Reviewer 1: If the 6th leaf is newly developed after defoliation, is 8 days enough for it to reach maturity? Furthermore, if LPI-6 is newly expanded, then the experiments deal with a leaf that has expanded under defoliation conditions. In other words, this is a development paper in addition to a physiology paper and is influenced by the developmental response to defoliation. This response can be quite profound. The developmental status of the leaf should be discussed in more detail.

Response: LPI-6 expanded during the period of recovery after partial defoliation. It is fair to ask whether the leaf had reached maturity by then. It was certainly a net source rather than a net sink. Based on leaf area data from previous work, LPI-6 was about 75% fully expanded, with LPI-8 being very nearly fully expanded. We have now clarified this in MM (lines 132-135) and Results (265-267). We hope this can address the reviewer's last request that the developmental status of the leaf should be discussed in more detail. We are not prepared to go beyond clarifying the status since the study was not conducted in order to draw developmental conclusions. Also, the points we make about leaf water content (addressed below) are consistent with intrinsic differences between RNAi leaves regardless of a differential defoliation effect on leaf expansion (now shown in new data of Figure 5). Panel B of Figure 2 was presented to make the point that even RNAi leaves that were unquestionably fully mature before defoliation exhibited higher post defoliation water contents than comparable wild type leaves. While there is no denying that there could be other developmental affects that we did not set out to study, the leaf water content response to partial defoliation was higher water content in RNAi than wild type leaves at final harvest.

Reviewer 1: The description of the leaves as new or already present is clearly explained in the experiments in Fig. 2. The simplest explanation for these results is that defoliation resulted in new leaves that were denser and had less water content, while the already-developed leaves had less water because defoliation led to root stress.

Response: Fig 2 actually was used to make a different point, that leaf water content was higher in RNAi than wild type leaves that expanded on partially defoliated plants, compared to equivalent leaves on 0-defoliation controls of each genotype. The reviewer states that newer leaves had lower water content than older leaves, but this is not what we showed in our data of Figure 2 (lines 290-295; 405-408; 425-427). There was no defoliation response difference between RNAi and WT for post-treatment-expanded leaves in the case of 100% defoliation. The larger point we intended to make from both panels of Fig 2 was that leaf water content was higher in RNAi than WT, not only in the leaves that expanded after the

partial defoliation treatments, but in leaves that were mature before defoliation. That includes leaves that were at LPI-10 or older at the time of defoliation, generally 25-35 leaves. We don't disagree with the reviewer's point in principal that root stress could have impacted newer leaves. However our data do not support the reviewer interpretation in this instance.   We will therefore keep our original interpretation of those data.

Reviewer 1: It is not sufficient to say (line 78) that the characteristics of the RNAi lines has been discussed. To understand the paper, the degree of downregulation and other pertinent information for these lines should be described in enough detail to interpret the paper.

Response: We have added that information to the paper in Materials and Methods (lines 100-103).

Reviewer 1: The experimental conditions represented by lines G and F should be explained in the figure legends.

Response: The experimental conditions were identical for all lines, WT, G and F. We have added additional text into the legends about experimental conditions.

Reviewer 1: What time of day were sugar and starch measurements made? If starch is making up for loss of sucrose in the transgenic lines, its hourly profile might differ from that of wt.

Response: Sampling for sugar and starch was always done at midday. We have added wording and two citations in Discussion lines (389-393) to incorporate the reviewers point.

Reviewer 1: The data on newly developed leaves, following defoliation, would be more meaningful if they included area measurements.

Response: We acknowledge the reviewer's point. Unfortunately the miss cannot be corrected for that experiment. What we can say is that there was nothing conspicuous to differentiate the leaf areas of WT and transgenic line leaves that expanded during the defoliation treatment. If something noticeable had developed in that regard during the experiment we would have been more than happy to have captured and reported on such a difference. This is not to suggest that there could not have been a subtle difference.

Reviewer 1: Line 18: I am not sure that one can say that tonoplast sucrose trafficking is more important for one function compared to another, based on quantitative response. Even a small quantitative response can have considerable effect. Perhaps some nebulous wording such as "proportionately influenced" would be better.

Response: Yes, agreed. We removed that sentence, but did not remove the point completely since that is a core matter of the paper. To respect the nuance we have referred to the point more circumspectly (lines 36-38; 44-46; 72-76).

Reviewer 1: The complex paragraph starting on line 39 is fine, once the reader gets the point. But it would help if that point were made at the beginning of the paragraph, not in the last sentence.

Response: Point taken. We substantially modified our Introduction in part because of unclear transitions such as the one pointed out here, and also because data are now added to the paper which justify further development of the Introduction. In addition, points raised below by reviewer 1 and later by reviewer 2 also led us to the conclusion that substantial changes had to be made in the Introduction. The paragraph originally starting at line 39 now is two paragraphs (lines 48-76). We hope that the Introduction makes its points in a more interesting way than in the original submission.

Reviewer 1: The discussion of OsSUT2 should be updated beyond Eom et al, 2012, especially the work from The Braun lab. Also, in the same sentence, the context of the term "passive transport" is not clear. It could refer to the short loading step or long-distance transport.

Response: We appreciate this point being made by the reviewer. In fact, we realized while making the fix that there were three related papers which we had left out, partly due to some tunnel vision on our part. We have now included Eom et al 2016; Leach et al 2017; Julius et al 2017. The reviewers will now note a substantial revision of the writing in Introduction especially with regard to updating (lines 25-46; 63-76).

Reviewer 1: Line 216, 221: "did not change" suggests leaf LPI-6 was already present at day 0. Perhaps it should read: "was not statistically different."

Response: Done. (Lines 267-268).

Reviewer: The last line of the Introduction is important because it states the goals of the paper. But it is a bit vague: "contributes to compensatory mechanisms." I would rewrite to make the objectives clearer.

Response: We have modified that sentence to lay things out less vaguely (lines 84-92).

Reviewer #2:
In the manuscript titled "Defoliation ‐ induced compensatory transpiration is compromised in SUT4 ‐ RNAi Populus", Scott Harding et al. provide novel insight on the potential function of sucrose transporter SUT4 in poplar. The topic carries high significance as poplar is an economically important species and understanding of carbon allocation at the molecular level might contribute to the biotechnological improvement of cultivated trees. The authors find that trees with reduced expression of SUT4 respond differently to defoliation compared to wild-type, mainly showing differences with regard to hydraulic conductivity.

A positive aspect of the manuscript is that all data is interpreted carefully and conclusions worded precisely. That being said, the last sentence of the abstract could be improved by a more direct formulation. The same applies to line 18 in the introduction, where the reader wonders how the importance of two hypothetical cases can be compared? Please reformulate.

Response: We understand the point and removed the subjective wording. The point is now addressed less subjectively in lines 44-46; 72-76.

Reviewer 2: Careful interpretation is highly appropriate as all data is somewhat indirect. Have the authors tried to determine subcellular sugar concentrations, for example with subcellular fractionation? Even if unsuccessful, inclusion of such results would be valuable. In the best case, it could provide direct prove for a difference in sugar concentration of vacuole and cytosol between wild-type and SUT4-RNAi plants. In the worst case, negative results (which, in case of subcellular fractionation are a realistic possibility) would keep other scientists from wasting time on such an approach. Obviously, subcellular sugar concentrations are not measurable with cellular resolution, as it would be wanted to validate the hypothesis put forward in the discussion section. However, even seeing a difference in the bulk leaf tissue would provide a more direct view of the possible background to the observations described here.

Response: As the reviewer suggests, sub cellular fractionation of metabolites can pose a serious technical challenge. We have performed such fractionation with these lines but we have found that the RNAi increase in sucrose leads to a spreading of the sucrose distribution in non-aqueous density gradients that impedes the extraction of new information beyond what we have reported for these lines in the past from bulk tissue analysis, basically higher sucrose and higher sucrose:hexose ratio than in WT source leaves. We only did non-aqueous metabolite partitioning for two independent replicates (leaves from separate plants) of one RNAi line and one wild type, which the reviewer probably recognizes, already entails an awful lot of very tedious work. The work was stopped once we realized the results would probably be equivocal, perhaps even with much more work to increase genotype and treatment representation. We choose not to include what data we did collect since it is possible that with a great deal of additional work, a more robust conclusion, positive or negative, might be supported. We do not want to come down one way or the other right now.

This said, we have developed new data which we now include in the manuscript to make the sucrose point in an indirect, but we believe, highly relevant manner. We carried out pressure-volume analysis of WT and RNAi leaves using a Schollander pressure bomb and methodologies now presented in Materials and Methods. The derived data are now presented in Figure 5 (lines 235-252 in Methods; 328-339 in Results; 416-427 in Discussion). The data show that the relationship between leaf turgor loss and leaf Ψw decrease differs between WT and RNAi. The data that we now present were derived from lab work during which tissue water loss per unit pressure increase was slower in RNAi than WT and followed a different kinetic-in other words, these are curvilinear plots from which the hydraulic parameters need to be derived. The raw plots also showed that the inflection in the curve at the turgor loss point differed between WT and RNAi. Turgor loss point itself did not differ between the lines. We only show derived data (storage water capacitance, C, and bulk modulus of elasticity, ε) that seemed sufficient for illustrating the existence of an important difference between RNAi and WT (Figure 5). The data are consistent with the interpretation that tissue water is more tightly held in RNAi than WT leaves. To gain a clue as to whether this seems to be more likely due to a difference in cell wall structure or alternatively to vacuolar sequestration of water due to increased sucrose sequestration in the central vacuole, we carried out TEM imaging, now included (Figure 6) (lines254-260 in Methods; 340-343 in Results; 416-427 in Discussion). The image data show increased central vacuole size in RNAi compared to WT leaf.

In a sense, the TEM data provide limited data pertaining to cell level resolution that would not have been achieved using non-aqueous partitioning, as pointed out by the reviewer. Epidermal cells were not as affected as palisade mesophyll cells in fully expanded leaves of the RNAi lines. We know from other

images which we would rather keep to ourselves for now, that the minor veins and bundle sheaths as well as bundle sheath extensions were not affected by SUT4-RNAi.

We have revised and toned down our speculation in Discussion to focus more narrowly on the implications of the new data in Figures 5 and 6.

In addition to this major recommendation, there are only two minor issues:

Reviewer 2: Line 33f. There are some microarray experiments that indicate a similar expression of PtaSUT1 and PtaSUT3 compared to PtaSUT4 in poplar leaves (see Fig. 2C in Xu et al. 2018, Plant Physiology 176).

Response: We are/were aware of that meta-analysis. We have now cited it in the manuscript (line 53) since it is a nice piece of work in many ways.

Reviewer 2: Lines 48-50. The drought-responsiveness of PtaSUT4 was also shown by Xu et al. (Plant Physiology, 2018). Fig. 4L therein shows a negative scaling of SUT4 with drought severity.

Response: The citations relating to Figure 4L in Xu et al were of our work, and we had a very hard time seeing how the data in 4L were derived from that work. In addition, we noticed that other graphs in Figure 4 of Xu et al treated OsSUT4 and ZmSUT4 as though they were the same class III tonoplast transporters actually discussed in the original papers. They were color highlighted while OsSUT2 and ZmSUT2 were not. OsSUT2 and ZmSUT2 are the actual class III tonoplast transporter orthologs in the original works. Furthermore, in looking at Eom et al 2011, those authors state clearly that the OsSUT2 promoter is not drought or abiotic stress responsive. Whether we are misunderstanding the meta-analysis, always a possibility, (I'll be the first to admit), or we are not, the presentation did not facilitate 'fact-checking' we would like to have done for our own comfort. Therefore we remain resistant to citing Xu et al 2018 in the context of the drought work with rice, maize, poplar. We apologize if we are simply missing something there. However, we do now cite that work elsewhere as mentioned
